# Exploring Middle Ear Pathologies in Adults with Diabetes Mellitus: A Scoping Review of Available Evidence and Research Gaps

**DOI:** 10.3390/ijerph22040503

**Published:** 2025-03-26

**Authors:** Ben Sebothoma, Katijah Khoza-Shangase, Gift Khumalo, Boitumelo Mokwena

**Affiliations:** 1 Department of Speech Pathology and Audiology, University of the Witwatersrand, Johannesburg 2050, South Africa; katijah.khoza-shangase@wits.ac.za (K.K.-S.); boitumelomokwena08@gmail.com (B.M.); 2 Centre for General Education, Faculty of Arts and Design, Durban University of Technology, Durban 2050, South Africa; giftk@dut.ac.za

**Keywords:** adults, conductive hearing loss, diabetes mellitus, middle ear pathologies, scoping review

## Abstract

Diabetes mellitus (DM) is a common chronic health condition, affecting millions of people worldwide, and its incidence is projected to increase by almost 50% in the next two decades. The effects of DM on the auditory system have been reported. However, there is limited evidence on the association between DM and middle ear pathologies. This scoping review aimed to map the available evidence and identify research gaps regarding DM and middle ear pathologies in the adult population. Five electronic databases, namely Scopus, CINAHL, MEDLINE, PubMed, and Web of Science, were searched using a combination of specific key terms. This review followed the guidelines stipulated by the Joanna Briggs Institute (JBI) methodology for scoping reviews and reporting using PRISMA Extension for Scoping Reviews (PRISMA-ScR): checklist and explanation. A thematic narrative analysis was used to synthesize key findings. Of the 1809 articles, only 2 articles met the inclusion criteria. Neither of these studies focused exclusively on middle ear pathologies in DM, but they did report incidental findings related to middle ear function. Available evidence suggests that middle ear pathologies may occur in individuals with DM, with a reported prevalence ranging from 3.1% to 19.6%. Otitis media with effusion and conductive hearing loss were common middle ear pathologies identified. Additionally, recent studies have provided new evidence suggesting ossicular joint changes in individuals with DM and a causal link between DM and acute suppurative otitis media. However, age-related hearing loss (presbycusis) and sensorineural hearing loss (SNHL) were more commonly associated with DM, with studies reporting a high prevalence of SNHL in younger to middle-aged adults with diabetes. This review highlights a significant research gap in the literature, as no studies directly investigated the relationship between DM and middle ear function as a primary focus. Further research is required to investigate this potential association using methodologies explicitly designed for middle ear assessment. While some evidence suggests a possible association, the lack of age-stratified analyses, imaging data, and comprehensive diagnostic testing limits the ability to draw strong conclusions. Further research incorporating age-based analyses, radiological assessments, and microbiome studies is needed to fully understand the potential impact of DM on middle ear health.

## 1. Introduction

Diabetes mellitus (DM) is one of the most common life-threatening chronic diseases, a chronic metabolic disorder characterized by elevated levels of blood glucose [1]. The elevation of blood glucose results from dysfunctional pancreas, which is unable to produce sufficient insulin, or the body’s inability to effectively use insulin produced by the pancreas [2,3]. Although there may be many types of DM [1], type 1 and type 2 are the most common types of diabetes diagnosed in many individuals. These types of DM reflect insulin-dependence due to dysfunction of pancreas (type 1) and insulin-resistance [3,4].

Many people around the world are living with DM [2]. It has reached epidemic proportions worldwide, with millions of individuals affected by the condition. The International Diabetes Federation estimates that approximately 537 million people are diagnosed and living with diabetes worldwide, with a projected prevalence expected to reach almost a billion (45% increase) by the year 2045 [5]. These high projections are concerning, especially in low- and middle-income countries (LMICs) due to shortages of health resources [6], high prevalence of other communicable diseases such as the human immunodeficiency virus and tuberculosis (TB), and other infectious diseases such as coronavirus, which are already putting a strain on the health system [7].

DM is a major public health concern in the 21st century. In South Africa, as in many other parts of the world, the prevalence of DM is on the rise. Factors such as urbanization, changing dietary habits, sedentary lifestyles, and genetic predisposition contribute to the increasing incidence of diabetes. It is estimated that a substantial portion of the adult population in South Africa is living with diabetes or is at risk of developing the condition. The prevalence of DM seems to be higher amongst the elderly population aged 60 years of age and above [8], particularly in low- and middle-income countries (LMICs) such as China, India, and Sub-Saharan countries [9,10]. While DM is growing worldwide, the prevalence of diabetes seems to be higher in the elderly population compared to the younger generation [11]. The risk of developing DM is particularly increased in adults aged 60 years of age and older due to a variety of factors that include genetic makeup, a long-life expectancy that causes a decrease in insulin secretion, and other lifestyle choices that contribute to central obesity [12,13].

DM is known to have systemic effects on various organs and systems in the human body, including the eyes, kidneys, nervous system, and cardiovascular system. However, the potential impact of diabetes on the auditory system, specifically the middle ear, has received less attention in the literature. The auditory system is a vital component of overall health and well-being. While the impact of DM on vision and cardiovascular health is well-documented, less is known about its potential influence on auditory health. The middle ear plays a crucial role in hearing, as it is responsible for conducting sound vibrations from the outer ear to the inner ear. Pathologies affecting the middle ear can result in hearing impairment and affect an individual’s quality of life. Studies reported that DM affects auditory organs because of macrovascular and microvascular problems, resulting in the thickening of the basilar membrane and stria vascularis capillaries [14]. Cochlear hair cells may develop lipid deposits because of lipid metabolic diseases, which can affect the cochlear neural cells and impair neural transmission [15]. An animal study indicates that DM may also affect mitochondrial function leading to hearing problems [16]. This study investigated the impact of diabetes on auditory function in a mouse model and found that diabetes-induced mitochondrial dysfunction contributed to cochlear synaptopathy and apoptosis of auditory cells. These findings suggest that diabetes may accelerate auditory system degeneration at a cellular level, providing insights into the mechanisms that could underlie hearing loss in humans with DM [16].

Recent evidence suggests that diabetes mellitus may influence middle ear function through structural and immunological mechanisms. A histopathological study by Shimura et al. [17] found significant ossicular joint changes in individuals with Type 2 DM, suggesting that diabetes-related metabolic alterations may impact middle ear mechanics. Additionally, a Mendelian randomization study by Kui et al. [18] demonstrated a causal association between Type 2 DM and an increased risk of acute suppurative otitis media (ASOM), indicating that individuals with DM may be more susceptible to middle ear infections due to immune dysfunction. These findings highlight the need to investigate the full spectrum of middle ear pathologies in individuals with DM and contextualize the current study’s findings.

The impact of DM on auditory health has been predominantly documented in relation to the vestibular and cochlear organs, with conditions such as sensorineural hearing loss being more commonly associated. However, despite its primary function as a conduit, the middle ear is not immune to systemic conditions that affect vascular and immune responses, as seen in DM. Changes in the vascular supply and immune dysfunction in DM may predispose the middle ear to pathologies such as otitis media or perforations, necessitating further exploration of this association.

A specific middle ear pathology that has been associated with DM is otosclerosis [19]. Otosclerosis is a bone remodeling disorder that affects the ossicles (stapes), leading to individuals with otosclerosis experiencing conductive hearing loss due to the fixation of the stapes in the oval window. Some studies have suggested a potential link between otosclerosis and DM, possibly due to the metabolic and vascular changes induced by diabetes that affect bone homeostasis [19]. Furthermore, hyperglycemia, a hallmark of DM, has been implicated in causing damage to small blood vessels throughout the body. Given the intricate vascular supply to the middle ear structures, it is plausible that hyperglycemia-related microangiopathy may affect the vascularization of the middle ear, potentially compromising its function.

The nature and prevalence of auditory pathologies associated with DM have been explored in the literature. Studies have indicated and reported that individuals diagnosed with DM are at an increased risk of hearing impairment [20,21]. Given the pathophysiology of DM, sensorineural hearing loss (SHNL) is reported to be the most common type of hearing impairment [22,23]. Bhat et al. reported the prevalence of SNHL to be 30% in individuals with DM [14]. Hlayisi et al. [24] found a slightly higher prevalence of hearing loss (55%) and SNHL (74%) in the adult population diagnosed with DM. Electrophysiological studies have also indicated that DM may affect the neural auditory pathway [25], leading to processing difficulties in the auditory and speech stimulus.

While there is existing literature on DM and hearing impairment, evidence regarding the association between DM and middle ear pathologies remains limited. The dearth of evidence in this area is concerning given that literature suggests that DM may affect the human immune system [26], resulting in individuals being prone to infections. Previous studies have indicated that individuals with immunocompromised systems due to medical conditions have also experienced various types and severity of middle ear pathologies [27,28,29,30].

Despite the growing prevalence of diabetes and its systemic effects on various organs, the relationship between diabetes and middle ear pathologies remains relatively unexplored. The scarcity of research in this area creates a significant knowledge gap that needs to be addressed. Understanding the potential impact of diabetes on middle ear health is not only relevant for academic and clinical purposes but also for the well-being of individuals living with diabetes. Therefore, exploring evidence regarding DM and middle ear function and pathologies may be crucial for early identification and intervention, preventing the sequelae of untreated pathologies. Furthermore, this study seeks to expand theoretical knowledge and understanding of the middle ear mechanics of individuals of different groups, particularly those with conditions that may alter the function of this system. Finally, this review intends to identify gaps that research can explore further.

## 2. Materials and Methods

This scoping review was conducted using the guidelines from the Joanna Briggs Institute (JBI) methodology for scoping reviews [31]. The scoping review was reported using PRISMA Extension for Scoping Reviews (PRISMA-ScR): checklist and explanation [32]. The review was not registered, and a protocol was not prepared or published.

### 2.1. Eligibility Criteria

The eligibility criteria were guided by the Population, Concept, and Context (PCC) framework that is stipulated in the JBI [31]. Table 1 outlines the eligibility criteria using the PCC framework. For studies to be included in this review, the following criteria had to be met: studies had to be published in a peer-reviewed journal in the English language, had to include an adult population aged 18 years and older, and had to report on middle ear pathologies using any test or measure of middle ear function. Studies were excluded if they were opinion papers, viewpoints, commentary, discussion papers, reviews, dissertations, thesis, research reports, and conference papers [33].

### 2.2. Information Sources and Search Strategy

Five electronic databases, viz, Scopus, PubMed, Medline, Cumulated Index to Nursing and Allied Health (CINAHL), and Web of Science (WOS), were searched. These databases were chosen based on the discussion and agreement between the researchers, and these databases were commonly used in the audiology literature [30,34], particularly in the literature exploring middle ear function and pathologies in various populations [28,30] and DM literature [35]. The search for articles on these databases was conducted in October 2023. The keywords used to search the databases included (“Middle ear pathology” OR “Middle ear disorder” OR “conductive hearing loss” OR “Hearing loss”) AND (“Diabetes”) AND (“Adults”).

### 2.3. Selection of Sources and Data Charting

All citations retrieved from the databases were first exported to Mendeley Reference Manager. Two authors (G.K. and B.M.) screened the titles and abstracts in accordance with the inclusion and exclusion criteria set for this review. Where a discrepancy existed between the two authors, the third author (B.S.) was consulted about the inclusion or exclusion of a particular article. A Microsoft Excel sheet was used to chart key information from the final included articles. These details included author/s, publication date, title, focus and aim, study design, measures used, context, outcome, and limitations.

### 2.4. Risk of Bias/Quality of the Selected Studies

The quality of the methodology of the selected studies of this current review was assessed using the adapted Newcastle–Ottawa Scale (Appendix A). This scale is appropriate for non-randomized studies.

## 3. Results

### 3.1. Study Selection

**Figure 1:**
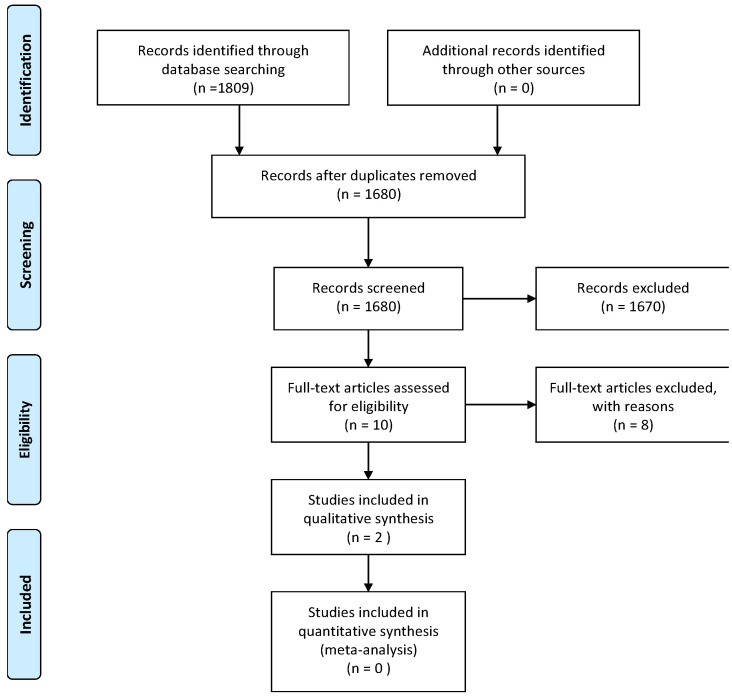
PRISMA flow diagram outlining the details of the search strategy.

Figure 1, below, shows the PRISMA flow diagram of articles included and excluded in this review. A total of 1809 articles were retrieved from the five databases. Following the removal of duplicates, 1680 articles were screened. During the title and abstract screening process, a total of 1670 articles were excluded. Common reasons for exclusion included studies on pediatric populations, studies focusing solely on sensorineural hearing loss or central auditory pathologies, and non-peer-reviewed articles. Of the 10 full-text articles assessed for eligibility, 8 were excluded. The reasons for full-text exclusions were primarily a lack of reporting on middle ear function, studies not meeting inclusion criteria, and articles published in non-English languages. Only two studies met the inclusion criteria. This reflects the significant scarcity of research specifically addressing middle ear function in individuals with diabetes mellitus (DM). Since this review follows the Joanna Briggs Institute (JBI) methodology for scoping reviews, its aim is to map existing evidence and identify research gaps, rather than to synthesize a large body of studies as in a systematic review. The limited number of included studies underscores the need for further high-quality research in this area.

Of the 1809 articles retrieved, only 2 articles were included for qualitative analysis (Table 2). However, it should be noted that neither of these studies directly focused on the relationship between DM and middle ear function. Instead, they broadly assessed auditory function, with middle ear pathologies being secondary findings. This highlights a significant gap in the literature specifically addressing middle ear function in the context of DM. Two studies included in this review specifically investigated the prevalence of sensorineural hearing loss (SNHL) in individuals with diabetes mellitus (DM). First, Hlayisi et al. [24], a South African study, compared 110 patients with DM to 82 non-diabetic controls, aged between 18 and 55 years. The findings revealed a significantly higher prevalence of disabling hearing loss in the diabetic group (35%) compared to the control group (8.5%). The hearing loss was predominantly bilateral, sensorineural, and affected high frequencies, suggesting a potential link between DM and auditory dysfunction. Second, Bhat et al. [14] conducted their study at a tertiary care center in Nepal, where they assessed 100 patients with DM, aged 40 to 70 years. The prevalence of hearing impairment was found to be 30%, with a higher occurrence in older patients and those with longer durations of diabetes. The hearing loss was primarily sensorineural and more pronounced at higher frequencies, indicating that age and duration of DM may contribute to auditory deficits. Both studies highlight a notable prevalence of SNHL among individuals with DM, particularly affecting high-frequency hearing thresholds.

### 3.2. Characteristics of the Studies

The two studies that were included for analysis in this scoping review were different studies that were conducted within the same context. These two studies were both conducted in Africa—Nigeria. Both studies specified the research design followed. One study employed a cross-sectional comparative study design [36], while the other study employed a case–control design [35]. Both studies reported on middle ear pathologies in adults diagnosed with DM. The total combined sample for the two studies included was 321 adults diagnosed with DM. With regard to the levels (or hierarchy) of evidence of the studies included, none of the studies were of the highest evidence (e.g., randomized control design).

Age is a significant factor in auditory dysfunction, particularly given the onset of presbycusis in mid-to-late adulthood. The two studies included in this review did not stratify their findings by age, making it difficult to distinguish whether middle ear pathologies observed in adults with DM were due to diabetes itself or an interaction between diabetes and age-related hearing decline. One study [36] reported a mean age of 47.6 years for participants, suggesting that the cohort was younger than the typical age range for presbycusis. However, without a detailed breakdown of hearing status by age groups, the influence of aging as a confounding factor remains unclear. Future studies should incorporate age-stratified analyses to determine whether middle ear pathologies in individuals with DM are distinct from age-related auditory degeneration.

### 3.3. Prevalence and Nature of Middle Ear Pathologies

Middle ear pathologies were common in adults diagnosed with diabetes and were reported to be relatively higher and more common compared to adults without diabetes. The prevalence of middle ear pathologies ranged from 3.1% to 19.6%. The lowest prevalence of middle ear pathologies was 3.1%, as determined by the air–bone gap of ≤15 dB HL [34], while the highest prevalence was otitis media with effusion (OME), with a prevalence of 19.6%, determined by clinical assessment methods [34]. The nature of middle ear pathologies seems to vary, with a significant number of individuals presenting with the severe form of middle ear pathologies, i.e., otitis media with effusion (19.6%) and perforated tympanic membrane (15.5%) [34].

### 3.4. Measures for Identification of Middle Ear Pathologies

Various measures were used to identify middle ear pathologies in adults diagnosed with DM. Pure tone audiometry using the air–bone gap principle was commonly used to identify middle ear pathologies [36,37]. While one study indicated which normative values (criteria) were used to determine whether there is an air–bone gap [37], the other study did not indicate the exact values to establish air–bone gaps [36]. Ordinary handheld otoscopy was also used by one study to assist in identifying middle ear pathologies. In another study, a clinical assessment of middle ear pathologies was performed using pneumatic otoscopy.

### 3.5. Quality of Studies

The two studies that were included in this review could be regarded as high quality based on the assessment scale (Appendix A).

## 4. Discussion

### 4.1. Overview of Main Findings

This review aimed to describe available evidence regarding middle ear pathologies and DM, and to establish research gaps in this area. The current scoping review demonstrates that while auditory pathologies associated with DM have been explored in the literature, there is a dearth of evidence on the association between middle ear pathologies and DM. Given that the pathophysiology of DM suggests an altered or dysfunctional immune system [38], leading to individuals being prone to infections, the dearth of evidence suggests the need for studies to provide an understanding of the middle ear mechanics in adults diagnosed with DM, and establish a tailormade early identification and intervention program.

While the vestibular and cochlear organs are more commonly recognized as being affected by DM, this review highlights the potential impact of DM on the middle ear. The middle ear, though primarily a conduit for sound transmission, may be affected by systemic conditions such as DM due to its reliance on adequate vascular supply and immune function. The findings of this review, though limited, suggest that middle ear pathologies such as otitis media with effusion may occur in diabetic populations. This highlights the importance of broadening the scope of research to include the middle ear when assessing the auditory effects of DM. The emerging literature supports the potential impact of diabetes mellitus on middle ear function. Shimura et al. [17] demonstrated that diabetes-induced metabolic changes can alter the ossicular joints, potentially leading to conductive hearing deficits. Additionally, Kui et al. [18] provided genetic evidence for a causal link between type 2 DM and acute suppurative otitis media, reinforcing the hypothesis that immune dysfunction in diabetes may contribute to middle ear pathologies. These findings complement the observations in this review by providing mechanistic insights into how diabetes may predispose individuals to middle ear dysfunction, warranting further investigation using advanced diagnostic tools such as tympanometry and imaging modalities like CT or MRI.

A critical finding of this review is the absence of studies directly investigating the relationship between DM and middle ear function. The two included studies broadly explored auditory function and reported on middle ear pathologies as incidental findings rather than as a primary focus. This distinction is important, as auditory function encompasses the entire auditory system, including the cochlea and central auditory pathways, while middle ear function specifically pertains to the mechanical conduction of sound. The lack of targeted research emphasizes a significant gap in the literature and highlights the need for studies that explicitly examine the potential impact of DM on middle ear health. The inclusion of only two studies may appear to weaken the conclusions of this review; however, this highlights the critical gap in the literature on middle ear pathologies in adults with DM. Unlike systematic reviews, which require a sufficient number of studies for meta-analysis, scoping reviews are designed to assess the breadth of available evidence and identify areas requiring further investigation. The fact that so few studies exist reinforces the need for targeted research on the relationship between DM and middle ear dysfunction, particularly using well-defined methodologies such as tympanometry, otoendoscopy, and radiological imaging.

Despite the dearth of evidence, the studies included in this review (*n* = 2) provide some insight into middle ear pathologies and DM. The studies indicate that middle ear pathologies are common in adults diagnosed with DM, and possibly higher in frequency. Although the middle ear is primarily recognized as a conduit for sound, the findings of this review suggest that it may also be susceptible to systemic conditions like DM. Expanding research to include middle ear pathologies alongside vestibular and cochlear dysfunctions will provide a more comprehensive understanding of the auditory effects of DM. While the sample size was relatively small, Adebola et al. found a higher prevalence of middle ear pathologies (19.6%) in adults diagnosed with DM [36]. The nature and severity of middle ear pathologies could only be established in one study [36], suggesting that adults diagnosed with DM may present with severe or advanced forms of middle ear pathologies such as perforated TM.

The difference in the prevalence and nature of middle ear pathologies between the studies included may be due to different measures and procedures used to identify the presence of middle ear pathologies. Middle ear pathologies were based on pure tone audiometry using air–bone gaps (≤15 dB HL) in one study, while the other study used both pure tone audiometry and clinical assessments such as pneumatic otoscopy. While pure tone audiometry can provide important clinical information on the middle ear system, it is not a direct measure of middle ear function. Therefore, it is not surprising that the prevalence of middle ear pathologies is higher in a study that utilized pneumatic otoscopy, which is a direct measure of middle ear pathologies [39] and is likely to identify more pathologies than pure tone audiometry.

While pneumatic otoscopy is an important clinical test for middle ear pathologies, the nature of middle ear pathologies demonstrates that this measure is more sensitive to more advanced forms of middle ear pathologies. Cho et al., and De Melker indicated that pneumatic otoscopy, while superior to ordinary single probe tone tympanometry, has higher sensitivity in identifying middle ear pathologies that have altered the mobility of the TM such as middle ear effusion [40,41]. Therefore, it is possible that subtle pathologies may have been missed, underrepresenting the prevalence and nature of middle ear pathologies in adults diagnosed with DM. Sensitive measures of middle ear pathologies, to understand the effects of DM on middle ear function, are needed [29].

The relationship between glucose levels, DM severity, and auditory pathologies remains underexplored. While the studies included in this review did not report a direct correlation between glucose levels or DM severity and the air–bone gap (ABG), this is a potential area for future research. Radiological imaging techniques such as computed tomography (CT) or magnetic resonance imaging (MRI) are essential in assessing the extent of chronic middle ear pathologies, particularly in understanding their relationship to systemic conditions like DM. However, neither study included in this review employed these imaging techniques, highlighting a gap in the methodology for studying middle ear pathologies in diabetic populations. Furthermore, recent evidence suggests that alterations in the microbiome, particularly in the oral, nasal, and ear regions, may contribute to the pathogenesis of otitis media. Given that the microbiome is significantly altered in individuals with DM (e.g., Xiao et al. [42]), future studies should explore the potential role of microbiome dysbiosis in the relationship between DM and middle ear pathologies (e.g., Frosolini and Lovato [41]). Investigating these aspects could provide deeper insights into the mechanisms underlying the observed associations and inform targeted interventions. The studies by Hlayisi et al. [24] and Bhat et al. [14] provide compelling evidence of an association between DM and SNHL. The higher prevalence of SNHL in individuals with DM, as reported in these studies, aligns with existing literature suggesting that diabetes may contribute to auditory dysfunction. However, the cross-sectional design of these studies limits the ability to infer causality. Further longitudinal research is necessary to elucidate the temporal relationship between DM and SNHL and to explore the underlying pathophysiological mechanisms.

While the review presents some important information on DM and middle ear pathologies, it certainly does not provide an unequivocal answer to the research question, and therefore does not provide trends on middle ear pathologies in adults diagnosed with DM. However, this review identifies an important research gap and emphasizes the need for extensive basic research that should employ various research designs, including those with high levels of evidence, with large sample sizes. These studies will assist in understanding middle ear function and pathologies in adults diagnosed with DM. These studies will also have clinical implications as they may help improve early identification and intervention of middle ear pathologies, especially in the context where DM is epidemic and continues to increase [2].

This review highlights a critical gap in the literature regarding the relationship between DM and middle ear function. While auditory dysfunction is well-documented in DM, there is no substantial evidence specifically addressing middle ear pathologies as a primary focus. The two studies included in this review provided limited insights, as they were not designed to investigate middle ear function in diabetic populations. Future research should prioritize studies that directly examine middle ear function using targeted methodologies, such as tympanometry or imaging, to elucidate any potential associations with DM.

Research on this topic holds significance on multiple fronts. Firstly, it contributes to bridging the existing gap in knowledge regarding the relationship between DM and middle ear pathologies. Secondly, the findings have the potential to enhance clinical practice by prompting early audiological assessments in individuals with DM. Moreover, understanding the implications of DM on auditory health can lead to improved patient care and the development of preventive strategies.

As the prevalence of DM continues to rise in South Africa and globally, investigating its potential impact on auditory health becomes increasingly important. The research outcomes may not only benefit individuals living with DM but also inform healthcare policies, preventive measures, and public health campaigns aimed at addressing the multifaceted health challenges posed by DM.

### 4.2. Limitations and Future Direction

While this review investigated an area that is not well explored, some limitations are presented. Firstly, this review included only studies that were published in peer-reviewed journals and no gray literature was included. Secondly, only studies published in English were included. The inclusion criteria used in this review might have excluded potentially important studies to expand this research. Lastly, due to the fact that scoping reviews are descriptive in nature and do not allow researchers to analyze data using various statistical methods, a definitive conclusion about a phenomenon cannot be made. Future research is needed to expand this area of investigation. Studies could utilize various research designs to explore the association between DM and middle ear pathologies. Future research should also consider including sensitive measures of middle ear pathologies such as wideband acoustic immittance (WAI), video otoscopy, and otomicrospy to determine the functioning of the middle ear system and middle ear pathologies of adults diagnosed with DM. Building on the findings by Shimura et al. [17] and Kui et al. [18], future research should explore how diabetes-related structural changes in the middle ear and increased susceptibility to infections contribute to auditory dysfunction. Investigating the interplay between metabolic changes, immune dysregulation, and middle ear pathologies could provide valuable insights into preventive and therapeutic strategies for individuals with DM. Prospective studies incorporating histopathological assessments, Mendelian randomization approaches, and advanced imaging techniques could bridge the existing knowledge gap and guide clinical management. Future research should also investigate the potential correlation between glucose levels, DM severity, and middle ear pathologies, including air bone gap (ABG). Radiological assessments such as CT and MRI could be integrated into studies to provide a comprehensive understanding of the structural changes in the middle ear associated with DM. Additionally, the emerging field of microbiome research offers a promising avenue for understanding the pathogenesis of middle ear conditions in diabetic populations. Alterations in the microbiome of the oral, nasal, and ear regions may play a role in the development of chronic middle ear conditions, particularly otitis media. Studies should examine the microbiome’s role in the relationship between DM and middle ear pathologies to enhance the depth and future impact of this research.

## 5. Conclusions

This review indicated that middle ear pathologies in adults diagnosed with diabetes mellitus exist. However, the findings highlight the limited evidence on the association between middle ear pathologies and diabetes mellitus. None of the studies directly explored this relationship. This limited evidence between middle ear pathologies and diabetes may pose a significant challenge in understanding the underlying mechanism, significantly hindering the development and implementation of early identification and intervention of middle ear pathologies in adults diagnosed with diabetes. Therefore, future research is required to investigate this potential association using methodologies that are appropriate and explicitly designed for middle ear assessment. The evidence from studies such as those by Hlayisi et al. [24] and Bhat et al. [14] highlights the importance of regular auditory assessments in individuals with DM. Early detection and management of hearing impairment can significantly improve the quality of life for these patients. Healthcare providers should be aware of the potential risk of SNHL in diabetic patients and consider integrating routine hearing evaluations into diabetes care protocols. This review highlights a significant research gap, as only two studies were found addressing middle ear function in individuals with DM. While this limits the ability to draw firm conclusions, it strongly suggests that this area is underexplored and warrants further investigation. Future studies should focus on high-quality, controlled research designs to better establish the relationship between DM and middle ear dysfunction, contributing to more evidence-based clinical management.

## Figures and Tables

**Table 1:** Eligibility criteria using the PCC framework.

Study Domain	Inclusion Criteria	Exclusion Criteria
Population (P)	Adult participants, aged 18 years and older	Pediatric population
Concept (C)	Middle ear function and pathologies in adults diagnosed with diabetes	Studies focusing on sensorineural hearing loss (SNHL) and/or central auditory pathologies
Context (C)	No restriction	No exclusion

**Table 2:** Details of study characteristics of each study included.

Author/s (date)	Publication Title	Publication Focus and Aim	Study Design	Measures Used	Study Participants	Context	Outcome	Limitations
Adebola et al. (2016) [36]	Otologic and audiologic characteristics of type 2 diabetics in a tertiary health institution in Nigeria.	To describe the pattern of otologic diseases and auditory acuities in type 2 diabetes mellitus patients, comparing this with those of non-diabetics and to explore the determinants of these patterns.	Cross-sectional and comparative study	Interviews, digital weight scale, BMI, plasma glucose levels, pneumatic otoscopy, pure tone audiometry.	The study included 97 patients diagnosed with type 2 diabetes and 90 non-diabetic patients.	Nigeria	There were 19.6% diagnosed with otitis media with effusion, 15.5% with perforated tympanic membrane, 14.2% of CHL (lower than the control).	Did not use sensitive measure of middle ear pathologies such as tympanometry. Pneumatic otoscopy is only sensitive for middle ear effusion.
Nwosu and Chime (2017) [37]	Hearing thresholds in adult Nigerians with diabetes mellitus: a case–control study.	To determine the prevalence, types and severity of hearing loss and associated factors in a hospital population of adult Nigerians with diabetes mellitus.	Case–control	Blood samples, urine samples, otologic examination including otoscopy, pure tone audiometry.	The study included 224 patients and 192 control participants. The patients comprised 112 males and 112 females (sex ratio = 1:1), whose mean age was 47.6 years (range: 26–80 years), diagnosed with diabetes.	Nigeria	Prevalence of CHL (air–bone gap of greater than 15 dBHL) was 3.1% in people with DM and 0% in control group.	Did not include sensitive measure of middle ear pathologies such as tympanometry.

## Data Availability

No new data were created or analyzed in this study.

## Appendix A: Summary of Quality Assessment of the Studies Using the Adapted Newcastle–Ottawa Scale

**Study**	**Selection** **(Max: 4 Stars)**	**Comparability** **(Max: 2 Stars)**	**Outcome** **(Max: 3 Stars)**	**Total Score** **(Max: 9 Stars)**	**Quality Rating**
Adebola et al. (2016) [36]	★★★★	★★	★★	8/9	High
Nwosu and Chime (2017) [37]	★★★	★★	★★	7/9	High

## References

[1] Banday M.Z., Sameer A.S., Nissar S. (2020). Pathophysiology of diabetes: An overview. Avicenna J. Med..

[2] da Silva J.A., de Souza E.C.F., Böschemeier A.G.E., da Costa C.C.M., Bezerra H.S., Feitosa E.E.L.C. (2018). Diagnosis of diabetes mellitus and living with a chronic condition: Participatory study. BMC Public Health.

[3] Gioacchini F.M., Pisani D., Viola P., Astorina A., Scarpa A., Libonati F.A., Tulli M., Re M., Chiarella G. (2023). Diabetes Mellitus and Hearing Loss: A Complex Relationship. Medicina.

[4] Katsarou A., Gudbjörnsdottir S., Rawshani A., Dabelea D., Bonifacio E., Anderson B.J., Jacobsen L.M., Schatz D.A., Lernmark Å. (2017). Type 1 diabetes mellitus. Nat. Rev. Dis. Primers.

[5] International Diabetes Federation (2022). Diabetes around the world in 2021. IDF Diabetes Atlas.

[6] Oleribe O.E., Momoh J., Uzochukwu B.S., Mbofana F., Adebiyi A., Barbera T., Williams R., Robinson S.D.T. (2019). Identifying Key Challenges Facing Healthcare Systems in Africa and Potential Solutions. Int. J. Gen. Med..

[7] Mokhele T., Sewpaul R., Sifunda S., Weir-Smith G., Dlamini S., Manyaapelo T., Naidoo I., Parker W.-A., Dukhi N., Jooste S. (2021). Spatial Analysis of Perceived Health System Capability and Actual Health System Capacity for COVID-19 in South Africa. Open Public Health J..

[8] Ko S.-H., Han K.D., Park Y.-M., Yun J.-S., Kim K., Bae J.-H., Kwon H.-S., Kim N.-H. (2023). Diabetes Mellitus in the Elderly Adults in Korea: Based on Data from the Korea National Health and Nutrition Examination Survey 2019 to 2020. Diabetes Metab. J..

[9] Animaw W., Seyoum Y. (2017). Increasing prevalence of diabetes mellitus in a developing country and its related factors. PLoS ONE.

[10] Lam A.A., Lepe A., Wild S.H., Jackson C. (2021). Diabetes comorbidities in low- and middle-income countries: An umbrella review. J. Glob. Health.

[11] Ramachandran A. (2014). Know the signs and symptoms of diabetes. Indian J. Med. Res..

[12] Chentli F., Azzoug S., Mahgoun S. (2015). Diabetes mellitus in elderly. Indian J. Endocrinol. Metab..

[13] Deshpande A.D., Harris-Hayes M., Schootman M. (2008). Epidemiology of Diabetes and Diabetes-Related Complications. Phys. Ther..

[14] Bhat N., Mahotra N.B., Shrestha L., Shrestha T.M., Tripathi P., Gupta M., Gurung S. (2021). Prevalence of hearing impairment in patients with diabetes mellitus at tertiary care center of Nepal. J. Appl. Biotechnol. Bioeng..

[15] Xipeng L., Ruiyu L., Meng L., Yanzhuo Z., Kaosan G., Liping W. (2013). Effects of Diabetes on Hearing and Cochlear Structures. J. Otol..

[16] Lyu A.-R., Kim T.-H., Shin S.-A., Kim E.-H., Yu Y., Gajbhiye A., Kwon H.-C., Je A.R., Huh Y.H., Park M.J. (2021). Hearing Impairment in a Mouse Model of Diabetes Is Associated with Mitochondrial Dysfunction, Synaptopathy, and Activation of the Intrinsic Apoptosis Pathway. Int. J. Mol. Sci..

[17] Shimura T., Yilmaz N.K., Rajan D., Cureoglu S., Monsanto R.D.C. (2024). Middle ear ossicular joint changes in Type 2 diabetes mellitus: A histopathological study. Laryngoscope.

[18] Kui L., Dong C., Wu J., Zhuo F., Yan B., Wang Z., Yang M., Xiong C., Qiu P. (2024). Causal association between type 2 diabetes mellitus and acute suppurative otitis media: Insights from a univariate and multivariate Mendelian randomization study. Front. Endocrinol..

[19] Kumari M.S., Meganadh K.R. (2016). Prevalence of Otological Disorders in Diabetic Cases with Hearing Loss. J. Diabetes Metab..

[20] Gupta S., Eavey R.D., Wang M., Curhan S.G., Curhan G.C. (2018). Type 2 diabetes and the risk of incident hearing loss. Diabetologia.

[21] Kim M.-B., Zhang Y., Chang Y., Ryu S., Choi Y., Kwon M.-J., Moon I.J., Deal J.A., Lin F.R., Guallar E. (2016). Diabetes mellitus and the incidence of hearing loss: A cohort study. Int. J. Epidemiol..

[22] Al-Rubeaan K., AlMomani M., AlGethami A.K., Darandari J., Alsalhi A., AlNaqeeb D., Almogbel E., Almasaari F.H., Youssef A.M. (2021). Hearing loss among patients with type 2 diabetes mellitus: A cross-sectional study. Ann. Saudi Med..

[23] Khoza-Shangase K., Pillay D., Moolla A. (2013). Diabetes and the audiologist: Is there need for concern regarding hearing function in diabetic adults?. S. Afr. J. Diabetes Vasc. Dis..

[24] Hlayisi V.-G., Petersen L., Ramma L. (2018). High prevalence of disabling hearing loss in young to middle-aged adults with diabetes. Int. J. Diabetes Dev. Ctries..

[25] AlJasser A., Uus K., Prendergast G., Plack C.J. (2019). Subclinical Auditory Neural Deficits in Patients with Type 1 Diabetes Mellitus. Ear Hear..

[26] Alves C., Casqueiro J., Casqueiro J. (2012). Infections in patients with diabetes mellitus: A review of pathogenesis. Indian J. Endocrinol. Metab..

[27] Khoza-Shangase K., Anastasiou J. (2020). An exploration of recorded otological manifestations in South African children with HIV/AIDS: A pilot study. Int. J. Pediatr. Otorhinolaryngol..

[28] Sebothoma B., Khoza-Shangase K. (2022). Middle ear status—Structure, function and pathology: A scoping review on middle ear status of COVID-19 positive patients. S. Afr. J. Commun. Disord..

[29] Sebothoma B., Khoza-Shangase K., Mol D., Masege D. (2021). The sensitivity and specificity of wideband absorbance measure in identifying pathologic middle ears in adults living with HIV. S. Afr. J. Commun. Disord..

[30] Sebothoma B., Maluleke M. (2022). Middle ear pathologies in children living with HIV: A scoping review. S. Afr. J. Commun. Disord..

[31] Peters M.D.J., Marnie C., Tricco A.C., Pollock D., Munn Z., Alexander L., McInerney P., Godfrey C.M., Khalil H. (2021). Updated methodological guidance for the conduct of scoping reviews. JBI Evid. Implement..

[32] Tricco A.C., Lillie E., Zarin W., O’Brien K.K., Colquhoun H., Levac D., Moher D., Peters M.D.J., Horsley T., Weeks L. (2018). PRISMA Extension for Scoping Reviews (PRISMA-ScR): Checklist and Explanation. Ann. Intern. Med..

[33] Oosthuizen I., Frisby C., Chadha S., Manchaiah V., Swanepoel D.W. (2023). Combined hearing and vision screening programs: A scoping review. Front. Public Health.

[34] Romli M., Timmer B.H.B., Dawes P. (2023). Hearing care services for adults with hearing loss in Malaysia: A scoping review. Int. J. Audiol..

[35] Pheiffer C., Wyk V.P.V., Turawa E., Levitt N., Kengne A.P., Bradshaw D. (2021). Prevalence of Type 2 Diabetes in South Africa: A Systematic Review and Meta-Analysis. Int. J. Environ. Res. Public Health.

[36] Adebola S.O., Olamoyegun M.A., Sogebi O.A., Iwuala S.O., Babarinde J.A., Oyelakin A.O. (2016). Otologic and audiologic characteristics of type 2 diabetics in a tertiary health institution in Nigeria. Braz. J. Otorhinolaryngol..

[37] Nwosu J.N., Chime E.N. (2017). Hearing thresholds in adult Nigerians with diabetes mellitus: A case–control study. Diabetes Metab. Syndr. Obes..

[38] Berbudi A., Rahmadika N., Tjahjadi A.I., Ruslami R. (2020). Type 2 Diabetes and its Impact on the Immune System. Curr. Diabetes Rev..

[39] Abbott P., Rosenkranz S., Hu W., Gunasekera H., Reath J. (2024). The effect and acceptability of tympanometry and pneumatic otoscopy in general practitioner diagnosis and management of childhood ear disease. BMC Fam. Pract..

[40] Cho Y.-S., Lee D.-K., Lee C.-K., Ko M.H., Lee H.-S. (2008). Video pneumatic otoscopy for the diagnosis of otitis media with effusion: A quantitative approach. Eur. Arch. Oto-Rhino-Laryngol..

[41] De Melker R.A. (1993). Evaluation of the diagnostic value of pneumatic otoscopy in primary care using the results of tympanometry as a reference standard. PubMed.

[42] Xiao E., Mattos M., Vieira G.H.A., Chen S., Corrêa J.D., Wu Y., Albiero M.L., Bittinger K., Graves D.T. (2017). Diabetes enhances IL-17 expression and alters the oral microbiome to increase its pathogenicity. Cell Host Microbe.

